# Preliminary Experience With a Novel Metallic Segmented Transcordal Stent Modified With Three-Dimensional Printing for Inoperable Malignant Laryngotracheal Stenosis

**DOI:** 10.3389/fonc.2021.619781

**Published:** 2021-07-26

**Authors:** Qungang Shan, Wei Huang, Ziyin Wang, Qingsheng Xue, Zhihong Shi, Jianping Zhou, Zhiyuan Wu, Xiaoyi Ding, Aiwu Mao, Mingyi Shang, Zhongmin Wang

**Affiliations:** ^1^ Department of Radiology, Ruijin Hospital, Shanghai Jiao Tong University School of Medicine, Shanghai, China; ^2^ Department of Anesthesiology, Ruijin Hospital, Shanghai Jiao Tong University School of Medicine, Shanghai, China; ^3^ Department of Otolaryngology & Head and Neck Surgery, Ruijin Hospital, Shanghai Jiao Tong University School of Medicine, Shanghai, China; ^4^ Department of Respiratory and Critical Care Medicine, Ruijin Hospital, Shanghai Jiao Tong University School of Medicine, Shanghai, China; ^5^ Department of Interventional Radiology, Tongren Hospital, Shanghai Jiao Tong University School of Medicine, Shanghai, China; ^6^ Department of Radiology, RuiJin Hospital/Lu Wan Branch, Shanghai Jiao Tong University School of Medicine, Shanghai, China

**Keywords:** laryngotracheal stenosis, malignancy, stent, vocal cord, three-dimensional printing

## Abstract

**Background:**

This study aims to assess the feasibility of a novel metallic segmented transcordal stent modified with three-dimensional (3D) printing for treating inoperable malignant laryngotracheal stenosis and the tolerability of the stent.

**Methods:**

This was a retrospective study. The stents were individually customized with the aid of 3D printing model based on the anatomic features of each patient’s airway. The stent was composed of two separate segments that corresponded to the larynx and the upper trachea. The stents were barrel-shaped at the proximal end to prevent migration. The proximal end of the stent was located slightly above the vocal cord. The technical and clinical success of stenting procedure, patient tolerability, and stent-related complications of patients were evaluated.

**Results:**

Ten patients with dyspnea caused by malignant laryngotracheal stenosis underwent implantation of such stents. Technical and clinical success of the stenting procedure were achieved in all patients. For all patients, basic communication in life could be maintained by speaking softly. During follow-up, one patient showed intolerance to the stent, and the stent was retrieved 2 weeks after stenting. Stent migration was found in one patient, and the position of the stent was readjusted. Granulation tissue proliferation was found in two patients and was treated with cryotherapy by bronchoscopy. There were no deaths associated with stenting.

**Conclusions:**

The individually customized metallic segmented transcordal stent is feasible and tolerable for patients with inoperable malignant laryngotracheal stenosis. The implantation of this stent may serve as a novel alternative treatment for patients who are not suitable for surgery or tracheotomy.

## Introduction

Laryngotracheal stenosis caused by malignancies that presents with severe dyspnea and a reduced quality of life is difficult to manage (1, 2). Although surgery is the treatment of choice, some patients are unfit for surgery due to their poor general condition or an advanced malignancy that has spread extensively into surrounding structures (3). Airway stenting has been widely used to treat benign or malignant airway stenosis (4). In inoperable patients, the implantation of laryngotracheal stents could serve as an alternative treatment that can provide immediate effects (5, 6). However, the complexity of the laryngeal and upper tracheal anatomy poses challenges for stenting, and the application of commercially available stents for laryngotracheal stenosis is limited due to stent-related complications such as stent migration and granulation tissue proliferation, especially when the stenosis is close to or involves the vocal cord (6, 7). Therefore, stents that match the anatomic and biomechanical features of the larynx and upper trachea are needed.

With the progress in computed tomography (CT) reconstruction and three-dimensional (3D) printing technology, a 3D model of patient-specific anatomy could be printed (8). Airway stents could be individually customized according to the anatomic features of the larynx and upper part of the trachea for each patient with the aid of the 3D model (8, 9). In addition, the 3D model could be used for preoperative planning to facilitate sufficient understanding of the anatomy (10, 11).

To overcome the disadvantages of commercial laryngotracheal stents, we developed a novel metallic segmented transcordal laryngotracheal stent that fits the laryngotracheal anatomy. The stent was individually customized with the aid of a 3D printing model. In this study, we aimed to assess the feasibility of the novel stent in treating inoperable malignant laryngotracheal stenosis and the tolerability of the stent. 

## Materials and Methods

### Patients

This retrospective study was approved by our institutional review board and written informed consent was waived. The clinical information of the patients was collected and analyzed. From October 2013 to March 2020, 16 patients underwent implantation of the novel metallic segmented transcordal stent modified with 3D printing due to severe dyspnea caused by malignant laryngotracheal stenosis. Prior to the stenting procedure, the institutional review board approved the use of the laryngotracheal stent and written informed consent was obtained from each patient. Six patients were excluded for the following reasons: (a) the patients underwent esophageal stenting (n=4); and (b) the patients had tracheoesophageal fistula (n=2). Finally, a total of 10 patients were included. The patients could not undergo surgical reconstruction due to the extensive invasion of the malignancy and their poor general condition. Tracheotomy was not performed for the following reasons: (a) the anterior part of the cervical trachea was covered by the tumor (n=6), and (b) esthetic considerations (n=4). After a discussion by the multidisciplinary team, which was composed of otolaryngologist, thoracic surgeon, respiratory physician, anesthetist and interventional radiologist, laryngotracheal stenting was determined as the treatment method.

### 3D Printing and Preoperative Plan

**Figure 1:**
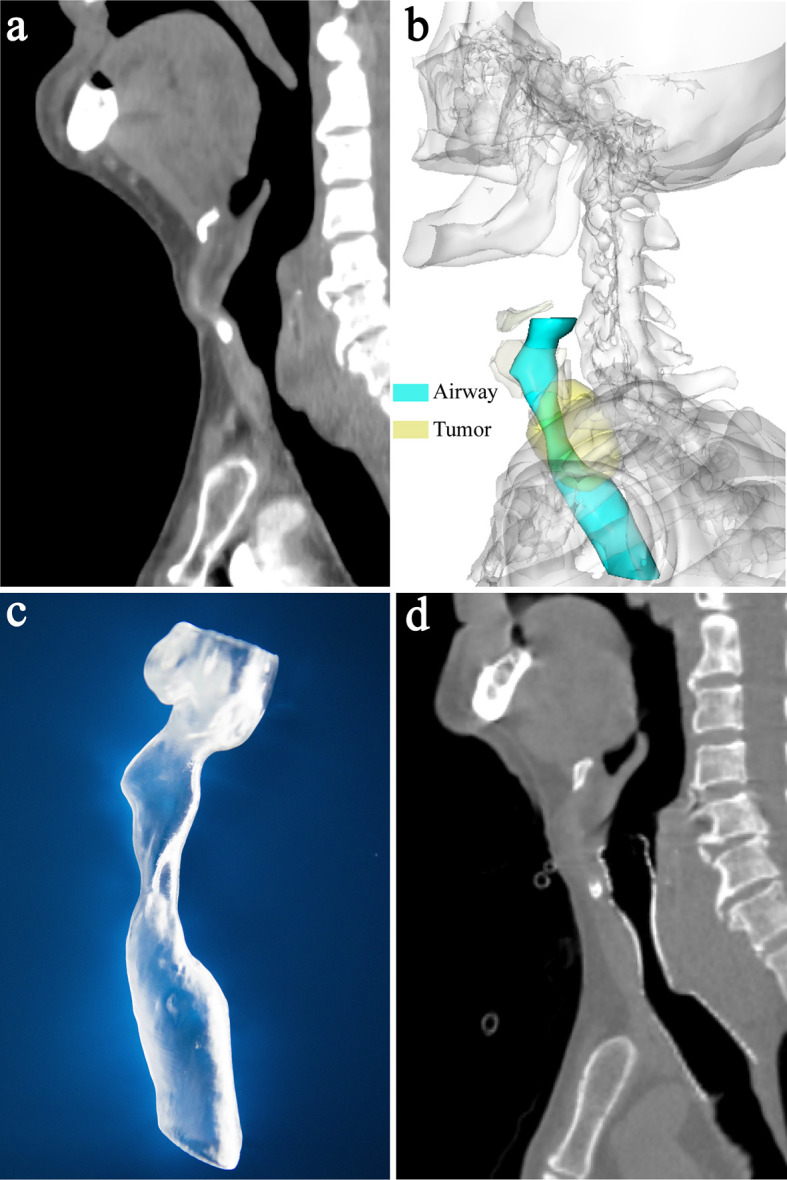
A 87-year-old man with laryngotracheal stenosis caused by thyroid cancer. **(A)** Sagittal CT showed subglottic and upper trachea stenosis caused by tumor compression before implantation of laryngotracheal stent. **(B)** 3-dimensional reconstruction image based on CT showed the stenotic subglottic area and upper trachea, and the relationship between them and surrounding structures. **(C)** The patient-specific model of larynx and upper trachea was made by 3-dimensional printer based on CT data. The diameter of the larynx and upper trachea, the extent and degree of stenosis, and the distance between the proximal end of the stenosis and vocal cord can be shown clearly on the model. **(D)** Sagittal CT showed that airway patency was maintained 1 month after stenting and the proximal end of the stent was located slightly above the vocal cord and below the epiglottis.

**Figure 2:**
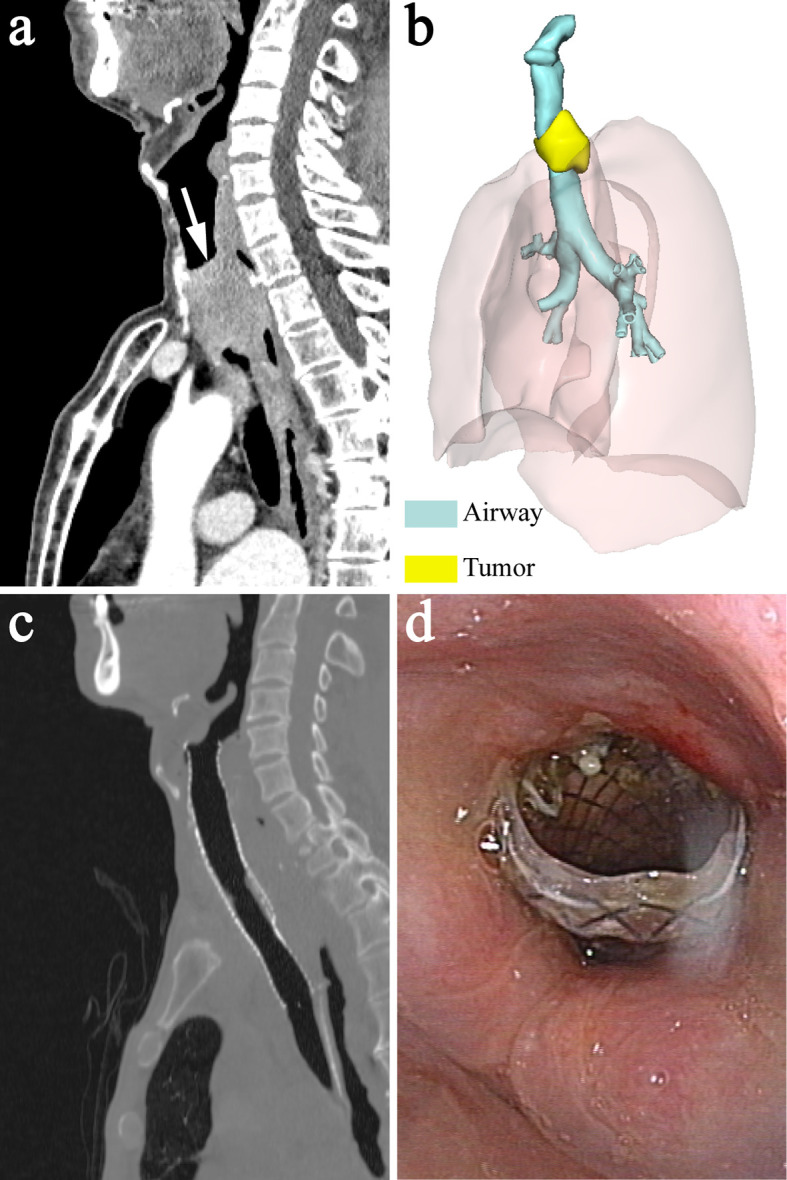
A 70-year-old man with upper tracheal stenosis caused by esophageal cancer. **(A)** Sagittal CT showed severe upper tracheal stenosis due to tumor compression before stenting (white arrow). **(B)** 3-dimensional reconstruction image showed the relationship between the stenotic upper trachea and surrounding tumor. **(C)** Sagittal CT showed that airway patency was restored 1 week after stenting and the proximal end of the stent was located slightly above the vocal cord and below the epiglottis. **(D)** Laryngoscopy 1 week after stenting showed that the proximal end of the stent was above the vocal cord, below the epiglottis and almost at the level of the arytenoid cartilages.

CT examinations of neck and thorax were performed and the parameters of CT scan were as follows: tube voltage of 120 kVp, tube current of 250 mA, and slice thickness of 1 mm. Multiplanar reconstruction of CT image was performed (**Figures 1A**, **2A**). 3D images of the larynx and trachea were reconstructed by computer-assisted design software (Vitaworks) using the raw CT data (**Figures 1B**, **2B**). Then, the 3D data was saved in stereolithographic format and was imported into the 3D printer (RS600, Union Tech), and 3D laryngotracheal models that were 1:1 matches with the larynx and upper trachea of each patient were printed layer by layer using photosensitive resins (**Figure 1C**).

The site, degree, range, and type of laryngotracheal stenosis, and the distance from the proximal end of stenosis to the vocal cord were determined by CT multiplanar reconstruction and the 3D printed model. Then, the target location for stent positioning was confirmed. The stenosis was graded by Myer-Cotton classification (2).

### Design of the Laryngotracheal Stent

**Figure 3:**
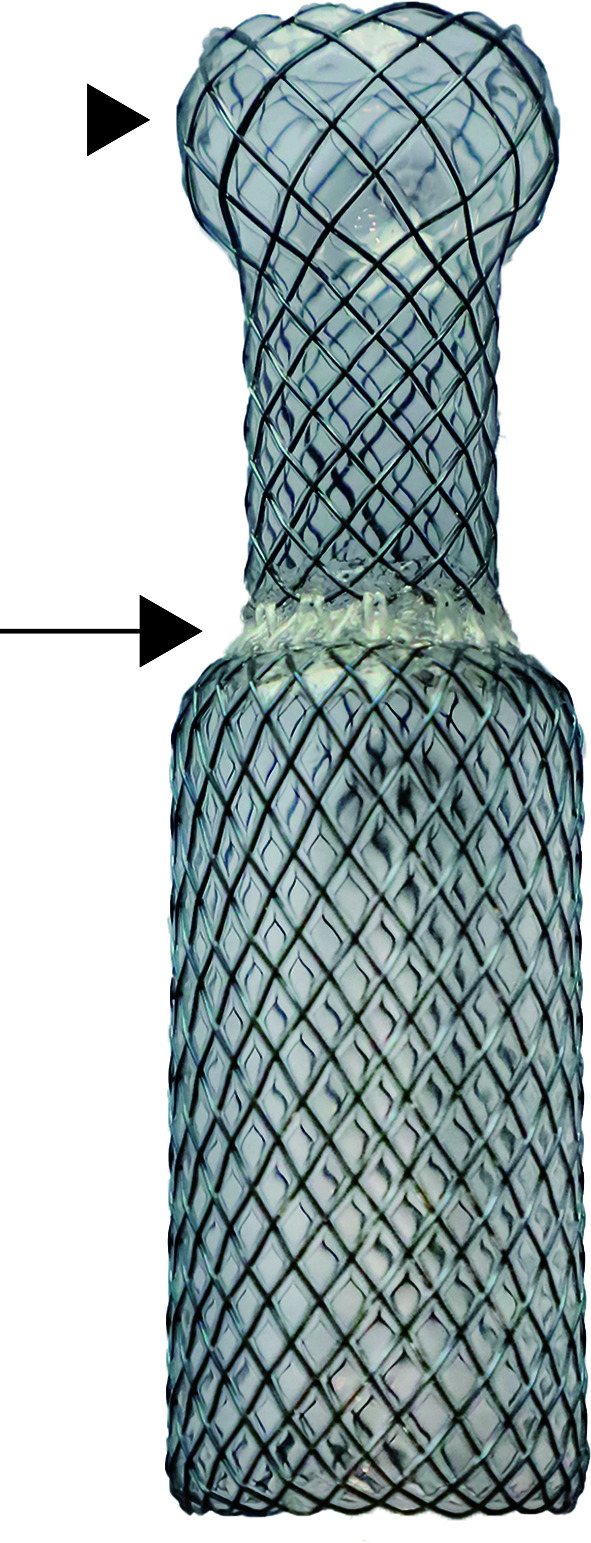
Image of metallic segmented transcordal stent. The stent was customized with the assist of the 3-dimensional-printed laryngotracheal model. The stent was composed of two separate parts that corresponded to the larynx and the upper trachea, and they were connected with poly(tetrafluoroethylene) (black arrow). The proximal end of the stent was barrel-shaped to prevent migration (black arrow head).

Based on the model, covered metallic self-expandable stents (Micro-Tech) were individually customized. The stents were woven with nickel titanium alloy wire. To reduce the risk of migration and accommodate the different diameters of the larynx and the upper trachea, a segmented stent design was applied. The segmented stent was composed of two separate parts that corresponded to the larynx and the upper trachea, and they were connected with poly(tetrafluoroethylene) (PTFE), a soft material, to reduce the interactions between the two segments. The diameters of the two segments were 10% larger than those of the corresponding parts of the larynx and the upper trachea. In addition, the proximal end of the stent was barrel-shaped to prevent migration (**Figure 3**). Ethylene oxide was used to sterilize the devices. The duration of stent manufacture was ranging from 3 to 4 days.

### Laryngotracheal Stenting Procedure

All laryngotracheal stenting procedures were performed in an operation room equipped with a digital subtraction angiography machine. The patients were placed in a supine position, and general anesthesia was administered. During the procedure, vital signs were closely monitored.

The glottis was adequately exposed with the laryngoscope, and then the guidewire was passed through the stenosis under the fluoroscopy guidance. After the guidewire exchange, the stent delivery system was advanced through a stiff guidewire. Afterwards, the laryngotracheal stent was released under the guidance of real-time fluoroscopy and bronchoscopy with the two ends of the stent extending past the stenotic segment, and the proximal end of the stent was located slightly above the level of the glottis and below the epiglottis.

After the stenting procedure, laryngoscopy, bronchoscopy, fluoroscopy and CT were immediately conducted to check the position and patency of the stent (**Video 1**).

### Follow-Up

CT and laryngoscopy were performed 1 week and 1 month after the stenting procedure to check the position and patency of the stent. Then, all patients received regular follow-up evaluations every 1 to 2 months. The patients were asked to return to the hospital for a re-examination if they felt discomfort during the period between the two follow-up evaluations. The pre- and postprocedure clinical manifestations of the patients were observed. The Hugh-Jones (HJ) classification was used to assess the dyspnea degree of the patients. Technical success of the stenting procedure was defined as accurate positioning of the stent across the stenotic segment with < 50% residual stenosis without major complications. Clinical success was defined as relief of obstructive symptoms. Patients were monitored for stent-related complications. Stents were retrieved by the retrievable thread if stent-related complications were observed or if the stents were no longer needed due to the reduced tumor burden. The follow-up information was obtained by reviewing the medical records or by directly contacting the patients or their family members by telephone.

### Statistical Analysis

Qualitative data are presented as numbers and percentages. Quantitative data are presented as the median with interquartile range (IQR). The pre- and postprocedure HJ classifications were compared using Wilcoxon tests for paired samples. Overall survival was estimated using the Kaplan-Meier method. The statistical analyses were carried out using SPSS 22.0 (IBM Corp). A P < 0.05 was considered statistically significant.

## Results

### Patient Characteristics

The median patient age at the time of the stenting procedure was 68.5 years (IQR: 62.75-81.75 years). All of the patients suffered from dyspnea due to laryngotracheal stenosis caused by malignancies, including esophageal carcinoma (n=6), laryngocarcinoma (n=1) and thyroid cancer (n=3). Six patients had vocal cord involvement caused by malignancies and had voice hoarseness. The other clinical manifestations included cough (n=5) and dysphagia (n=2). The baseline patient characteristics are shown in **Table 1**.

**Table 1:** Baseline characteristics of patients.

Characteristics	Patients (n=10)
Age (years) ^a^	68.5 (62.75-81.75)
Gender	
Male	7
Female	3
Etiology	
Esophageal carcinoma	6
Thyroid cancer	3
Laryngocarcinoma	1
Location of stenosis	
Upper trachea	2
Upper trachea+subglottic area	3
Upper trachea+subglottic area+ glottic region	5
Type of stenosis	
Extraluminal	7
Mixed	3
Hugh-Jones classification ^a^	5 (4-5)

a Reported as median (IQR).

### Technical and Clinical Outcomes

Arterial oxygen saturation was maintained at > 97%, and no complications occurred throughout the procedure. Technical success was achieved in all patients. Airway patency was recovered following sufficient expansion of the stent, which was immediately confirmed by laryngoscopy, bronchoscopy, fluoroscopy and CT. The proximal end of the stent was slightly above the level of the vocal cord and below the epiglottis in all patients (**Figure 1D** and **Figures 2C, D**). All patients showed clear symptomatic relief of dyspnea after stent deployment. The patients had significantly lower HJ classifications after the procedure than before the procedure [2 (IQR: 2-3) *vs* 5 (IQR: 4-5); P = 0.004].

### Follow-Up

There were no deaths associated with stenting. All patients underwent chemotherapy and/or radiotherapy after the procedure.

The median follow-up time was 150 days (IQR: 67.5 - 215 days). All patients felt a foreign body sensation, and 4 of the 10 patients felt pain in their larynx, especially when swallowing, coughing and speaking. One of the patients did not tolerate the discomfort caused by the stent, and the stent was retrieved 2 weeks after stenting. All other patients could tolerate the stent, and the symptoms improved or disappeared in 3-7 days. Voice hoarseness was found in all patients. Specifically, 6 patients exhibited voice hoarseness before the stenting procedure due to involvement of the vocal cord, and a voice change was observed after the procedure. The other 4 patients without involvement of the vocal cord developed voice hoarseness after the procedure. For all patients, basic communication in life could be maintained by speaking softly. During follow-up, 4 of the patients presented with mild aspiration that resolved spontaneously, and severe aspiration that required treatment did not occur. In 2 patients, restenosis was caused by granulation tissue formation around the proximal end of the stent 3 and 5 months after stenting procedure, respectively. They were treated with cryotherapy by bronchoscopy. Mucus retention was found in 3 patients and was treated by sputum suction through bronchoscopy. Stent migration was found in 1 patient, and the position of the stent was readjusted under bronchoscopy and fluoroscopy. The median overall survival time for these patients was 180 days.

## Discussion

In the current study, we presented the design of a novel metallic segmented laryngotracheal stent customized with the aid of 3D printing and reported our preliminary experience with a series of 10 patients with malignant laryngotracheal stenosis treated with implantation of such stents that were precisely positioned above the level of the vocal cord. The results of this study indicated that the stent was feasible and tolerable in the treatment of laryngotracheal stenosis caused by malignancies when located above the level of the glottis.

Laryngotracheal stenosis can be caused by various factors, including tumor invasion and trauma (1, 2). The management of malignant laryngotracheal stenosis is difficult (1, 2). Although surgical reconstruction is the preferred treatment option for malignant laryngotracheal stenosis, some patients lose the chance for surgery due to extensive tumor invasion or poor general status. For patients who are unfit for surgery, alternative approaches include tracheotomy and stent implantation (3, 5, 7). As a minimally invasive therapeutic approach, the implantation of laryngotracheal stents, including Montgomery T-tubes and silicone and metallic stents, have been used in the treatment of laryngotracheal stenosis (6, 12, 13). This approach could also provide an alternative solution for patients who are unfit or unwilling to undergo tracheotomy. In this study, most patients tolerated the stent, and no patients died of stent-related complications, but the complications were not insignificant. Maintaining a balance between the risks and benefits of transcordal stenting is crucial to achieving optimal results for each patient and requires a multidisciplinary discussion in the management of patients.

3D printing technology is becoming increasingly important in the medical field. The 3D printed anatomic model generated from CT imaging can demonstrate complex laryngotracheal anatomy and is helpful for both airway stent design and preoperative planning (10, 11). The diameter of the larynx and upper trachea and the location, length and degree of stenosis were different among patients (14). Therefore, commercial stents may not match the airway well and thus cannot meet the anatomic and biomechanical needs of each patient, especially in the larynx and upper trachea, which are sensitive to foreign bodies (5). To achieve a good match, a personalized stent is required. In this study, the features of the patient’s anatomy and stenosis were reflected by the 3D printed laryngotracheal model, and then the stent was individually tailored accordingly. Therefore, a stent with a better match to the airway than a commercial stent could be obtained, which helps to relieve patient discomfort and reduce the risk of complications. In addition, the model could be used as a tool during preoperative planning to facilitate accurate positioning of the stent. Previous studies have reported that 3D-printed laryngotracheal models showed better performance than CT images and endoscopy in terms of determining the extent of glottic-subglottic airway stenosis (10). The distance between the proximal end of the stenosis and vocal cord could be precisely shown by the 3D model, which could facilitate clinical decision making for each patient. Furthermore, the 3D model could also be used for resident teaching and patient education (10, 15). However, it should be noted that the major limitation of customized stents is the time required to manufacture the stent. For this reason, the application of customized stents cannot be generalizable to emergency patients.

Precise positioning of the stent is very important in improving the patient’s tolerance and reducing the risk of stent-related complications (6, 7). For subglottic stenosis, the upper margin of the stent should be kept at least 1 centimeter away from the vocal cord to reduce vocal cord stimulation (6, 7, 16). Otherwise, subglottic edema and granulation tissue formation slightly below the vocal cord may occur, especially if the stent migrates in this region, which could cause patient discomfort and even dyspnea (5). However, for stenosis involving the glottis or the subglottic area less than 1 centimeter below the vocal cord, the stent should be placed above the cord to sufficiently cover the stenosis and avoid complications (6, 7, 17). Therefore, the distance from the proximal end of the stenosis to the vocal cord determines the location to place the stent. Previous studies have reported the application of transcordal stents, including Montgomery T-tubes, straight silicone stents (Dumon stents) and hourglass-shaped silicone stents, and promising results have been obtained (6, 17, 18). In this study, the stents were placed with the proximal end slightly above the vocal cord and below the epiglottis, and no severe aspiration occurred, which was similar to previous studies (6, 18, 19). This is because normal closure of the epiglottis and elevation of the larynx could be guaranteed through precise positioning of the stent in this region (6, 7). In this study, the stent was released under the combined guidance of bronchoscopy and fluoroscopy in real time and therefore precise positioning of the stent was confirmed.

A major challenge of stenting for laryngotracheal stenosis is the high risk of migration. The diameter of the larynx was slightly shorter than that of the upper trachea, and thus, a conventional straight stent could not match both the larynx and upper trachea (14). Undersize diameter of the stent would increase the risk of migration, and conversely, oversizing the diameter of the stent would lead to discomfort and granulation tissue proliferation. In addition, the larynx moves during the processes of phonation, coughing, swallowing and neck movements, which may cause migration (20). To prevent stent migration, a segmented design and barrel-shaped proximal end were employed in the modified stent reported in this study. Unlike conventional stents, the diameters of the two parts of the segmented stent were determined according to those of the larynx and the upper trachea, ensuring that the stent would fit the anatomical characteristics of the larynx and upper trachea better. In addition, the upper part of the stent could move with the larynx, and the movement magnitude of the lower part of the stent was relatively small due to the soft connection between the two parts. Most patients in this study showed good tolerance to the segmented stents. Segmented designs have been used in esophageal stents, and the stimulation of the stent to the wall of esophagus could be reduced, especially for circuitous esophageal lesions (21). Moreover, the barrel-shaped proximal end of the stent plays a positive role in fixing the stent as well. Several kinds of stent flanges, including flared, dumbbell and barrel shapes, have been used in esophageal stents to prevent stent migration, and the anti-migration force of the stent with a barrel-shaped flange was the largest (22, 23). In this cohort of patients, stent migration occurred in 1 patient during follow-up, which was similar to a previous study reported on transcordal stents (6). Different non-slip designs preventing stent migration have been used for T-tube and silicone stents in previous studies. The side arm of the T-tube could effectively reduce the risk of migration. However, for patients with extensive tumor invasion of anterior wall of trachea or recurrent tracheostoma infections, implantation of a T-tube is undesirable (24). In addition, some patients are unwilling to undergo T-tube implantation for esthetic reasons (24). The stent reported in this study did not have the aforementioned limitations and could be used in these conditions without tracheotomy. Regarding straight silicone stents, migration cannot be prevented by the stud alone (20). The external fixation apparatus of silicone stents is effective in preventing migration in patients with laryngotracheal stenosis (20, 25, 26). However, this option is not suitable for patients with a pathological area between vocal cord and cricoid. The stent in this study did not need external fixation and could be implanted with a simpler procedure.

The other stent-related complications could be managed effectively. All patients in this study showed dysphonia, but they were able to maintain communication with others by speaking softly, which is consistent with previous studies on transcordal stents (20, 25, 26). Although granulation tissue proliferation occurred at the end of the stent in patients, this complication did not lead to fatal airway stenosis due to timely detection during follow-up and could be treated by ablation.

The limitations of this study are the retrospective nature, the small sample size, and the lack of a control group. In addition, the etiology of laryngotracheal stenosis was mixed. Further studies should be performed to validate our conclusions.

This preliminary study indicated that metallic segmented transcordal stents individually customized with the assist of 3D printed model are feasible and tolerable for patients with inoperable malignant laryngotracheal stenosis. The implantation of this stent may serve as a novel alternative treatment for patients who are not suitable for surgery or tracheotomy.

## Data Availability Statement

The raw data supporting the conclusions of this article will be made available by the authors, without undue reservation.

## Ethics Statement

The studies involving human participants were reviewed and approved by the institutional review board of RuiJin Hospital/Lu Wan Branch. Written informed consent for participation was not required for this study in accordance with the national legislation and the institutional requirements.Written informed consent for participation was not required for this study in accordance with the national legislation and the institutional requirements.

## Author Contributions

QS, WH, ZMW, and MS designed the research and supervised the report. QS, WH, ZMW, MS, ZYW, QX, ZS, JZ, ZYW, XD, and AM performed the research and analyzed the data. QS and WH wrote the manuscript. All authors contributed to the article and approved the submitted version.

## Funding

Supported by the Shanghai key specialty construction project (No. ZK2015A22 and No. ZK2019A02); Clinical key specialist construction project of Shanghai municipal health commission (Interventional Radiology [No. shslczdzk06002] & 3D Printing [No. shslczdzk07002]; and Shanghai municipal commission of health and family planning (No. 201640087).

## Conflict of Interest

The authors declare that the research was conducted in the absence of any commercial or financial relationships that could be construed as a potential conflict of interest.

## Publisher’s Note

All claims expressed in this article are solely those of the authors and do not necessarily represent those of their affiliated organizations, or those of the publisher, the editors and the reviewers. Any product that may be evaluated in this article, or claim that may be made by its manufacturer, is not guaranteed or endorsed by the publisher.

## Supplementary Material

Supplementary Video 1 Preoperative examination, stenting procedure, and follow-up. The video (wmv) showed the preoperative 3-dimensional CT reconstruction, stenting procedure under the guidance of real-time fluoroscopy and bronchoscopy and CT and laryngoscopy follow-up after stenting.
